# Mfn2 Regulates High Glucose-Induced MAMs Dysfunction and Apoptosis in Podocytes *via* PERK Pathway

**DOI:** 10.3389/fcell.2021.769213

**Published:** 2021-12-20

**Authors:** Yun Cao, Zhaowei Chen, Jijia Hu, Jun Feng, Zijing Zhu, Yanqin Fan, Qiaoxuan Lin, Guohua Ding

**Affiliations:** ^1^ Division of Nephrology, Renmin Hospital of Wuhan University, Wuhan, China; ^2^ Nephrology and Urology Research Institute of Wuhan University, Wuhan, China

**Keywords:** DKD, podocyte, Mfn2, MAMs, ER stress, apoptosis

## Abstract

The endoplasmic reticulum (ER) stress and mitochondrial dysfunction in high glucose (HG)-induced podocyte injury have been demonstrated to the progression of diabetic kidney disease (DKD). However, the pathological mechanisms remain equivocal. Mitofusin2 (Mfn2) was initially identified as a dynamin-like protein involved in fusing the outer mitochondrial membrane (OMM). More recently, Mfn2 has been reported to be located at the ER membranes that contact OMM. Mitochondria-associated ER membranes (MAMs) is the intercellular membrane subdomain, which connects the mitochondria and ER through a proteinaceous tether. Here, we observed the suppression of Mfn2 expression in the glomeruli and glomerular podocytes of patients with DKD. Streptozotocin (STZ)-induced diabetic rats exhibited abnormal mitochondrial morphology and MAMs reduction in podocytes, accompanied by decreased expression of Mfn2 and activation of all three unfolded protein response (UPR) pathways (IRE1, ATF6, and PERK). The HG-induced mitochondrial dysfunction, MAMs reduction, and increased apoptosis *in vitro* were accompanied by the downregulation of Mfn2 and activation of the PERK pathway. Mfn2 physically interacts with PERK, and HG promotes a decrease in Mfn2-PERK interaction. In addition, Mfn2-silenced podocytes showed mitochondrial dysfunction, MAMs reduction, activation of PERK pathway, and increased apoptosis. Conversely, all these effects of HG stimulation were alleviated significantly by Mfn2 overexpression. Furthermore, the inhibition of PERK phosphorylation protected mitochondrial functions but did not affect the expression of Mfn2 in HG-treated podocytes. Therefore, this study confirmed that Mfn2 regulates the morphology and functions of MAMs and mitochondria, and exerts anti-apoptotic effects on podocytes by inhibiting the PERK pathway. Hence, the Mfn2-PERK signaling pathway may be a new therapeutic target for preventing podocyte injury in DKD**.**

## Introduction

With the increase in incidences of diabetes mellitus (DM), DKD has become a public health issue, which is the leading cause of chronic kidney disease (CKD) and end-stage renal disease (ESRD) (Umanath and Lewis, 2018). Furthermore, glomerular lesions are regarded as the most common renal pathological changes in DKD (Carrara et al., 2020; Jefferson et al., 2008).

Podocytes, are the terminally differentiated glomerular epithelial cells, regarded as a crucial component in maintaining the integrity of the glomerular filtration barrier (Torban et al., 2019; Wang et al., 2018). Podocyte injury and loss have been well demonstrated to be correlated with proteinuria and renal function deterioration, thus, they are considered to be the key events in the progression of DKD, but their pathological mechanisms have not yet been elucidated.

Mitochondria and ER are two interconnected functional units in the eukaryotic cells. The contact points through which ER tethers to mitochondria are termed MAMs. Recent studies proved that mutual regulation between ER and mitochondria, and the existence of MAMs provides a new way to explain these phenomena (Hamasaki et al., 2013; Lewis et al., 2016). Meanwhile, the mitochondrial dysfunction and ER stress have drawn the attentions, which are closely related to podocyte injury and apoptosis in DKD (Chen et al., 2020; Cybulsky, 2010; Fan et al., 2019). Given the crucial roles of ER stress and mitochondrial dysfunction, it is imperative to reveal the underlying mechanisms through which MAMs evolve and affect the injury and apoptosis of podocyte in diabetic kidneys.

Mfn2 is a GTPase that mediates OMM fusion by forming homologous or heterologous dimers to link two adjacent mitochondria with Mfn1 (Escobar-Henriques and Anton, 2013). Mfn2 loss-of-function significantly reduces mitochondrial fusion efficiency and induces mitochondrial fragmentation. Mfn2 is found to be highly located at the MAMs and regulates ER and mitochondrial functions, such as calcium balance, protein synthesis, mitochondrial fusion and fission, mitophagy, and inflammatory response (Bernard-Marissal et al., 2019; Hernandez-Alvarez et al., 2019; Yuan et al., 2020). Patients with obesity and T2DM have reduced Mfn2 expression in skeletal muscle (Theurey and Rieusset, 2017). Metabolomics analysis of the urinary exosomes and renal biopsy tissues of patients with DKD revealed abnormal expression of a variety of proteins and regulatory factors related to mitochondrial metabolism (Sharma et al., 2013). Mfn2 overexpression sustained mitochondrial functions and increased the survival rate in lipopolysaccharide (LPS) -treated human kidney2 (HK2) cells (Liu et al., 2017).

Mfn2 overexpression also ameliorates ER stress-induced cell death, while Mfn2 knockout upregulates the UPR markers *in vivo* and *in vitro* (Munoz et al., 2013; Ngoh et al., 2012; Sebastian et al., 2012). UPR is the first activated response after ER stress occurs and is responsible for coordinating the cellular transcription and translation, improving protein folding of unfolded proteins, and accelerating the degradation of misfolded proteins. Inositol requiring protein1(IRE1), protein kinase RNA-like ER kinase (PERK), and activating transcription factor6 (ATF6) are the three UPR sensors (Walter and Ron, 2011). Once the misfolded or unfolded proteins accumulate and disturb ER homeostasis, the activated UPR sensors activate the follow-up pathways. The interaction between Mfn2 and PERK have proposed that occurs in the MAMs (de Brito and Scorrano, 2008; Verfaillie et al., 2012). In favor of this view, both Mfn2 and PERK are regarded to be located at the membranes.

In preliminary experiments, we found that decreased expression of Mfn2 along with the activation of the PERK pathway and increased apoptosis in podocytes under HG conditions. In this study, we reconfirmed the above results. Further, we found that HG-induced Mfn2 downregulation and PERK pathway activation accompanied MAMs reduction and mitochondrial dysfunction *in vivo* and *in vitro*. However, the roles of Mfn2 and PERK, as well as their potential regulatory effects on the alteration of MAMs and mitochondria, the injury and apoptosis of HG-treated podocytes, have not been well investigated.

Therefore, this study aimed to explore the role of the Mfn2-PERK signaling pathway in the injury and apoptosis of podocytes initiated by HG-induced MAMs and mitochondrial dysfunction, and further elucidated its potential molecular mechanism.

## Materials and Methods

### Human Renal Biopsy Samples

Renal biopsy samples from patients with biopsy-proven DKD (n= 6) (4 males and 2 females; age 42–66 years; mean age 57.0 ± 9.27 years) were obtained from the Division of Nephrology, Renmin Hospital of Wuhan University (Wuhan, China). Para-carcinoma tissues from patients without diabetes who underwent tumor nephrectomies (n = 6) (3 males and 3 females; age 40–63 years; mean age 53.2 ± 8.40 years) were used as controls and were obtained from the Division of Pathology, Renmin Hospital of Wuhan University (Wuhan, China).

Histologic examination and biochemical analysis (urine albumin-to-creatinine ratio <30 mg/g) showed no features of DKD apart from solitary renal cell carcinoma. Investigations were carried out on the principles of the Declaration of Helsinki. Informed consent was obtained from all patients included in this study. Experiments were performed according to the guidelines of Wuhan University, which the Research Ethics Committee of Renmin Hospital of Wuhan University approved.

### Animals

A total of twelve 8-week-old male Sprague–Dawley rats were randomly divided into the diabetic group (6 rats) and the control group (6 rats). The type I diabetes rat model was established by the intraperitoneal injection of streptozotocin (STZ, 65 mg/kg of body weight; S0130, Sigma-Aldrich, United States). The control rats received 0.1 mol/L citrate buffer (pH = 0.45) (Chen et al., 2020). Blood glucose was measured after administration of STZ. The rats with a blood glucose level exceeding 16.7 mM were considered diabetic. The blood glucose and body weight of rats were recorded at the end of 0, 4, 8, 12, and 16 weeks. In addition, 24-h urine samples were collected at the end of 12 and 16 weeks to evaluate total urine proteins (UTP) at 24 h. All rats were sacrificed at 12 or 16 weeks post-STZ injection, and the kidneys were collected and used for subsequent testing.

### Cell Culture and Treatments

Conditionally immortalized human podocytes (HPC) were provided by Dr. Moin A. Saleem (Academic Renal Unit, Southmead Hospital, Bristol, United Kingdom) and cultured in RPMI 1640 medium (SH30809.01, HyClone, United States) supplemented with 10% fetal bovine serum (FBS, 10100; Gibco, United States), penicillin G (100 U/ml) -streptomycin (100 μg/ml) (15140122; Life, United States), 1×insulin transferrin-selenium (ITS, 41400045; Life, United States) at 33°C for proliferation. When the density of HPC reached 70–80%, the cells were transferred to 37°C with an ITS-free medium for differentiation (Fan et al., 2019; Hu et al., 2019; Lin et al., 2020). The differentiated HPC were cultured using different mediums (normal glucose, N = 5 mM d-glucose; mannitol, MA = 5 mM D-glucose+20 mM mannitol; high glucose, HG = 25 mM d-glucose) for 24 h or 36 h; The PERK-inhibited HPC were treated with 20 µM PERK phosphorylation inhibitor (GSK2656157; Selleck, United States).

### Cell Transfection

Cell transfection was conducted using the X-tremeGENE HP DNA Transfection Reagent (Rh-06366 236 001; Roche, Swit). A total of 2 × 10^5^ HPC were seeded into six-well plates, transfected with Mfn2-Myc plasmid (Addgene) or shRNA-Mfn2 plasmid (PIEE248070577; Shanghai GenePharma Co., Ltd.) for Mfn2 overexpression or knockdown, and transfected with pcDNA3.1 plasmid (Addgene) or scrambled-shRNA plasmid (Shanghai GenePharma Co., Ltd.) as control. The HPC were incubated under normal cultured conditions for 36–48 h after adding the complexes of plasmids (1 μg/ml) and transfection reagent (1 μL/ml). The HPC transfected with Mfn2-Myc/pcDNA3.1 plasmid (Addgene) were cultured using HG (25 mM d-glucose) medium for 24 h after transfection 36–48 h.

### Transmission Electron Microscopic Analysis

The renal cortex of rats and HPC were fixed in 2.5% glutaraldehyde to further observe the ultrastructure of glomerular podocyte, mitochondrial morphology, MAMs integrity of glomerular podocyte and HPC (Fan et al., 2019). A transmission electron microscope (TEM; Hitachi, Japan) was used to observe all of the microscopic images.

The aspect ratio of mitochondria and MAMs contact points were assessed using ImageJ software. The percentage of the mitochondrial membrane in contact with ER within a 50 nm range was measured and normalized to the mitochondrial perimeter, as previously described (Tubbs et al., 2018).

### Histological Analysis

The frozen renal biopsies from patients and the frozen kidney sections from rats were stained with Hematoxylin and Eosin (H&E) and Periodic acid-Schiff (PAS) reagents to observe the histopathological changes of the kidney. An upright microscope (Olympus, Japan) was used to observe all of the microscopic images.

### Western Blot Analysis

For Western blot analysis, total protein from the isolated glomeruli or HPC was extracted with RIPA buffer (P0013; Beyotime, China) mixed with protease inhibitor cocktail (p8340; Sigma-Aldrich, United States), phosphatase inhibitor phosSTOP™ (49068370001; Sigma-Aldrich, United States), and PMSF (ST506; Beyotime, China). The extractions were centrifuged at 13,000 rpm at 4°C for 5 min, then the supernatants were mixed with loading buffer and boiled at 100°C for 10 min. Equal amounts of protein were separated by 8–12% SDS-PAGE and transferred onto PVDF membranes (Millipore, United States). After blocking with 5% skimmed milk at room temperature for 1 h, the membranes were incubated with primary antibodies (Fan et al., 2019). The following primary antibodies were used in this study: Mfn2 (ab123773; 1:1,000; Abcam, United Kingdom); cleaved caspase3 (ab32042; 1:1,000; Abcam, United Kingdom); CHOP (sc-7351; 1:500; Santa Cruz, United States); Bcl2 (ab32124; 1:1,000; Abcam, United Kingdom); Bax (60267-1-lg; 1:1,000; Proteintech, United States); IRE1α (#3294; 1:1,000; CST, United States); ATF6β (15794-1-AP; 1:1,000; Proteintech, United States); p-PERK (#3719; 1:1,000; CST, United States); PERK (sc-377400; 1:500; Santa Cruz, United States); Myc-tag (#562; 1:1,000; Medical & Biological Laboratories CO. LTD., Beijing, China); GAPDH (sc-32233; 1:2000; Santa Cruz, United States). Secondary antibodies conjugated to Horseradish peroxidase (HRP) (ANT019s, ANT020s, 1:10000; Antgene, Wuhan, China) were used at room temperature for 1 h. The membranes were soaked with enhanced chemiluminescence solution (ECL, G2014, Servicebio, Wuhan, China), and the blots were further observed with an X-ray machine (Bio-Rad, United States).

### Coimmunoprecipitation

Coimmunoprecipitation was carried out to verify the interaction between Mfn2 and PERK. First, the lysates of HPC cultured in 10 cm^2^ plates were subject to immunoprecipitation with 3–5 µl PERK or Mfn2 antibody. The immunoprecipitates were blotted with Mfn2 (1:1,000; Abcam, United Kingdom) or PERK (1:500; Santa Cruz, United States) antibody at 4°C overnight and then centrifuged at 13,000 rpm at 4°C for 5 min to get the supernatants. After being mixed with loading buffer boiled at 100°C for 10 min, the protein samples were processed for Western blot analysis (Ma et al., 2018).

### Immunohistochemistry

The frozen renal biopsies from patients and the frozen kidney sections from rats were fixed with 4% paraformaldehyde for 30 min. After block with 5% bovine serum albumin (BSA, ANT101L; Antgene, China) at room temperature for 1 h, the sections were then incubated with the primary antibody (Fan et al., 2019). The Mfn2 (1:200; Abcam, United Kingdom) antibody was used as the primary antibody in this study. All microscopic images were observed using an upright microscope (Olympus, Japan).

### Immunofluorescence Assay

The immunofluorescence assay analyses using the frozen renal biopsies from patients, the frozen kidney sections from rats and the cell climbing films were performed as previously described. The following primary antibodies were used in this study: Mfn2 (1:200; Abcam, United Kingdom); WT1 (NB110-60011, 1:50; Novus, GER); Synaptopodin (65194, 1:50; Progen, GER); TOM20 (sc-17764; 1:50; Santa Cruz, United States); Calreticulin (ab92516; 1:100; Abcam, United Kingdom); PERK (1:50; Santa Cruz, United States). The Alex Fluor 488/594 donkey anti-rabbit/Mouse IgG (HL) (ANT023, ANT024, ANT029, ANT030; 1:100; Antgene, Wuhan, China) were used as secondary antibodies. The nuclei were stained with an anti-fluorescence quencher containing DAPI (ANT063; Antgene, Wuhan, China). A confocal microscope (Olympus, Japan) was used to observe all of the microscopic images. The Pearson’s correlation coefficients (PCC) have been calculated in immunofluorescence assay of TOM20 and CRT, which with a value between 1 and −1, was used to quantitative colocalization between two proteins (1 means perfect correlation; −1 means completely excluded; 0 means no relationship) (Schober et al., 2018).

### ROS Generation, Mitochondrial Membrane Potential, and ATP Production Analyses

Mitochondrial ROS generation measured using the DCFH-DA fluorescent probe (S0033S; Beyotime, China); Mitochondrial membrane potential (MMP) was evaluated by JC-1 staining (C2003S; mitochondrial membrane potential assay kit with JC-1; Beyotime, China); The ATP Assay Kit (S0027; Beyotime, China) was used to measure the ATP levels.

### Apoptosis Assay

The glomerular podocyte apoptosis of rats was analyzed by co-staining with TUNEL (C1086; One Step TUNEL Apoptosis Assay Kit, Beyotime, China) and Synaptopodin antibody (1:50; Progen, GER).

The apoptosis rate of HPC was measured by flow cytometry with Annexin V-PE/7AAD double staining (#556547, Annexin V-PE Apoptosis Detection Kit I, BD Pharmingen, United States).

### Statistical Analyses

All experiments were performed at least three times independently. All data were summarized as the means ± SD and analyzed using GraphPad Prism5 (GraphPad Software, Inc.). One-way or two-way analysis of variance (ANOVA) was applied to analyze data for comparisons between groups. The *p* < 0.05 was considered significant.

## Results

### Clinical and Histopathological Changes and Mfn2 Expression in Diabetic Rats

**FIGURE 1:**
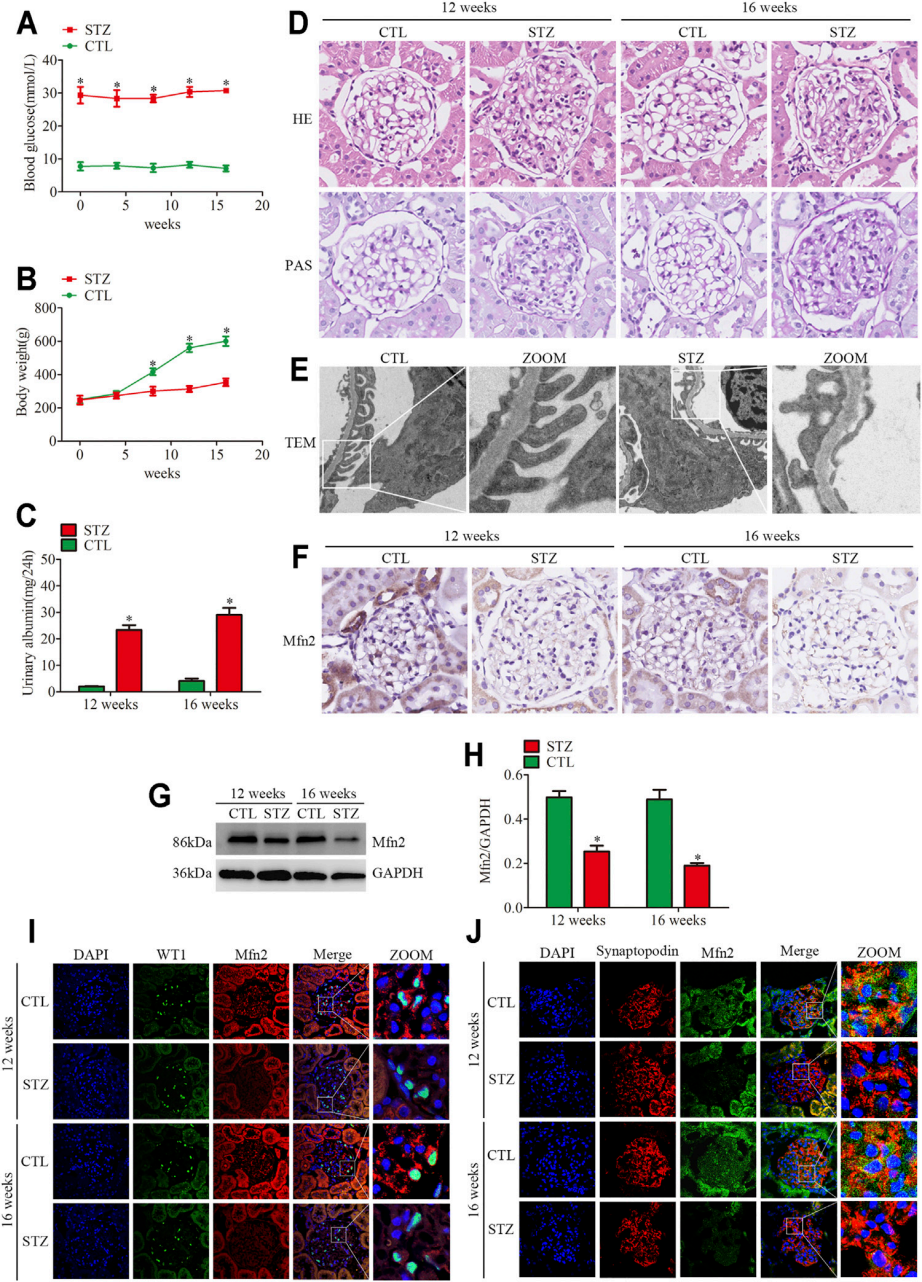
Histopathological changes and Mfn2 expression in diabetic rats. **(A)** Blood glucose levels per group (n = 3). *p < 0.05 relative to control at the same time point. **(B)** Bodyweight per group (n = 3). *p < 0.05 relative to control at the same time point. **(C)** Quantitative analysis of 24 h urine total proteins (UTP) of rats at 12 or 16 weeks post-STZ injection (n = 3). *p < 0.05 relative to control at the same time point. **(D)** Histological changes in rats after STZ injection were determined by HE and PAS staining (original magnification, ×600). **(E)** The TEM images of the glomerular ultrastructure of rats at 16 weeks post-STZ injection (original magnification, ×3,000). **(F)** Representative images of immunohistochemical staining of Mfn2 in glomeruli per group (original magnification, ×600). **(G,H)** Representative Western blots of glomerular Mfn2 expression and quantitation per group (n = 3). *p < 0.05 relative to control. **(I,J)** Representative images of immunofluorescent staining of Mfn2, WT1 or Synaptopodin and DAPI in glomeruli per group (original magnification, ×600). CTL, control; STZ, streptozotocin; HE, Hematoxylin and Eosin; PAS, Periodic acid-Schiff; TEM, transmission electron microscope; Mfn2, Mitofusin2.

The STZ-treated rats developed hyperglycemia, and their body weight decreased compared to the control group (Figures 1A,B). Differences in 24-h urine total proteins (UTP) were observed between the two groups, suggesting an impaired renal function in the diabetic rats (Figure 1C). In addition, both HE and PAS staining demonstrated that the diabetic animals presented evident mesangial matrix expansion compared to the controls (Figure 1D). Furthermore, the transmission electron microscopic (TEM) analysis revealed significant fusion of podocyte foot process in diabetic rats (Figure 1E). Collectively, these findings indicated glomerular damage and podocyte injury in diabetic rats.

Even though MAMs dysfunction has been reported to be associated with metabolic syndrome, including DKD (Yang et al., 2021), the *in vivo* studies on MAMs of podocytes under hyperglycemia conditions are limited. The TEM analysis of glomerular podocytes demonstrated mitochondrial fragmentation and MAMs reduction in the diabetic rats compared with those in the controls, which indicated elongated mitochondria and abundant contact points localized at the interface of the ER and outer mitochondrial membrane (Supplementary Figures 1B–D). In addition, we performed the triple immunofluorescent staining of TOM20 (mitochondrial marker), Calreticulin (CRT, ER marker), and DAPI to evaluate ER-mitochondrial interactions in glomeruli. The results indicated reduced ER-mitochondrial interactions in the diabetic rats (Supplementary Figure 1A).

The expression of Mfn2 was evaluated to clarify the mechanism of mitochondrial fragmentation and MAMs reduction in podocytes. Compared with the controls, the glomeruli from diabetic rats showed decreased Mfn2 levels (Figures 1F–H). The triple immunofluorescent staining of Mfn2, WT1 or Synaptopodin (podocyte marker) and DAPI was conducted to better observe the Mfn2 expression alterations. The results indicated that Mfn2 was located at the cytoplasm, and the Mfn2 expression was reduced in the podocytes of diabetic rats (Figures 1I,J). These results verified that hyperglycemia-induced Mfn2 expression decreases *in vivo*. Altogether, the results revealed that hyperglycemia-induced reduction of Mfn2 expression was related to glomerular damage and podocyte injury in DKD.

### Histopathological Changes of Glomeruli and Mfn2 Expression in Patients With DKD

**FIGURE 2:**
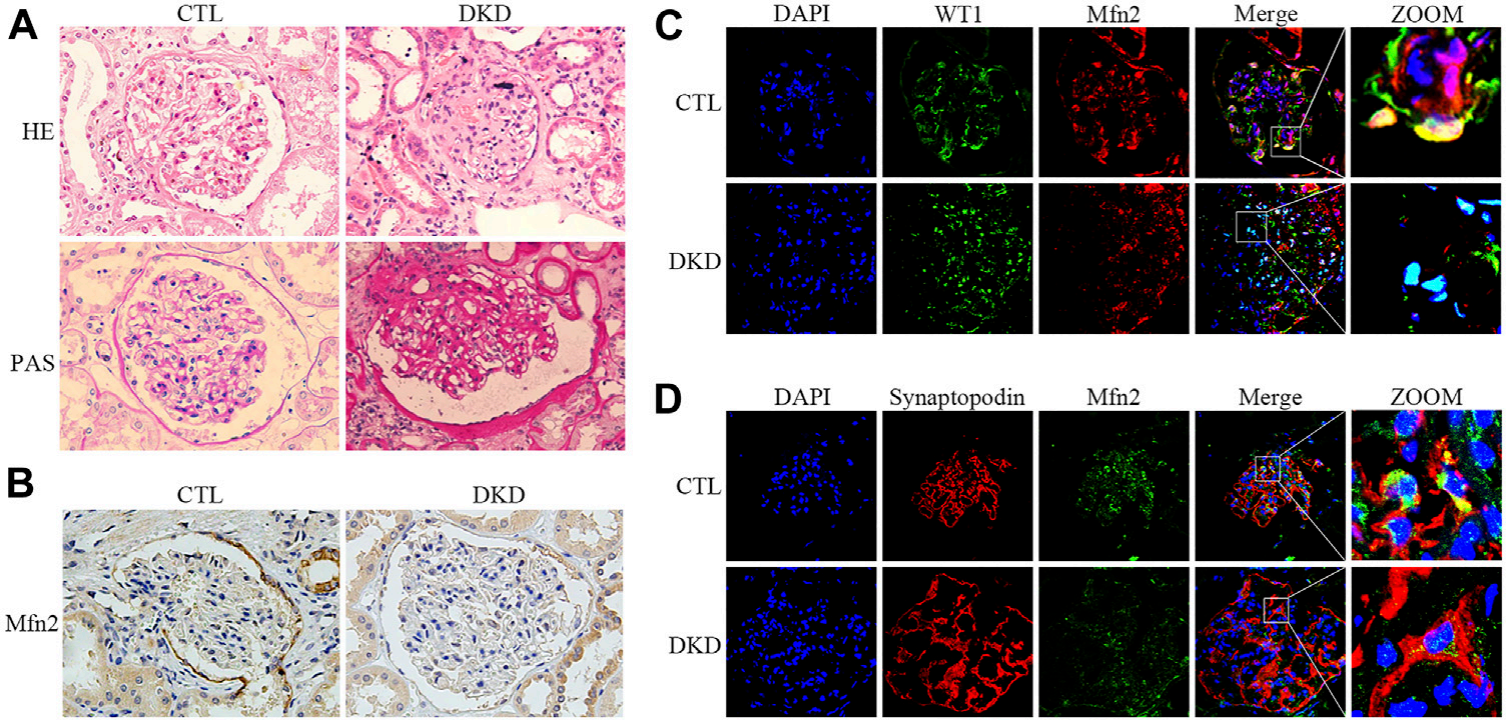
Histopathological changes and Mfn2 expression in patients with DKD. **(A)** Histological changes in patients with DKD were determined by HE and PAS staining (original magnification, ×400). **(B)** Representative images of immunohistochemical staining of Mfn2 in glomeruli per group (original magnification, ×400). **(C,D)** Representative images of immunofluorescent staining of Mfn2, WT1 or Synaptopodin and DAPI in glomeruli per group (original magnification, ×600). CTL, control; DKD, diabetic kidney disease.

Renal pathological changes such as extracellular matrix deposition were observed in patients with DKD through HE and PAS staining (Figure 2A). As with the tendency of Mfn2 expression in the glomeruli of diabetic rats, Mfn2 was also reduced in the glomeruli of patients with DKD (Figure 2B). The triple immunofluorescent staining of Mfn2, WT1 or Synaptopodin, and DAPI was performed to evaluate Mfn2 expression in glomerular podocytes. A dramatic reduction in the Mfn2 levels was detected in the podocytes of patients with DKD relative to those of healthy individuals (Figures 2C,D). The immunofluorescent staining of TUNEL, Synaptopodin, and DAPI showed increased podocyte apoptosis in patients with DKD (Supplementary Figure 2).

### Decreased Mfn2 Expression and MAMs Reduction in HG-Treated Cultured Podocytes

**FIGURE 3:**
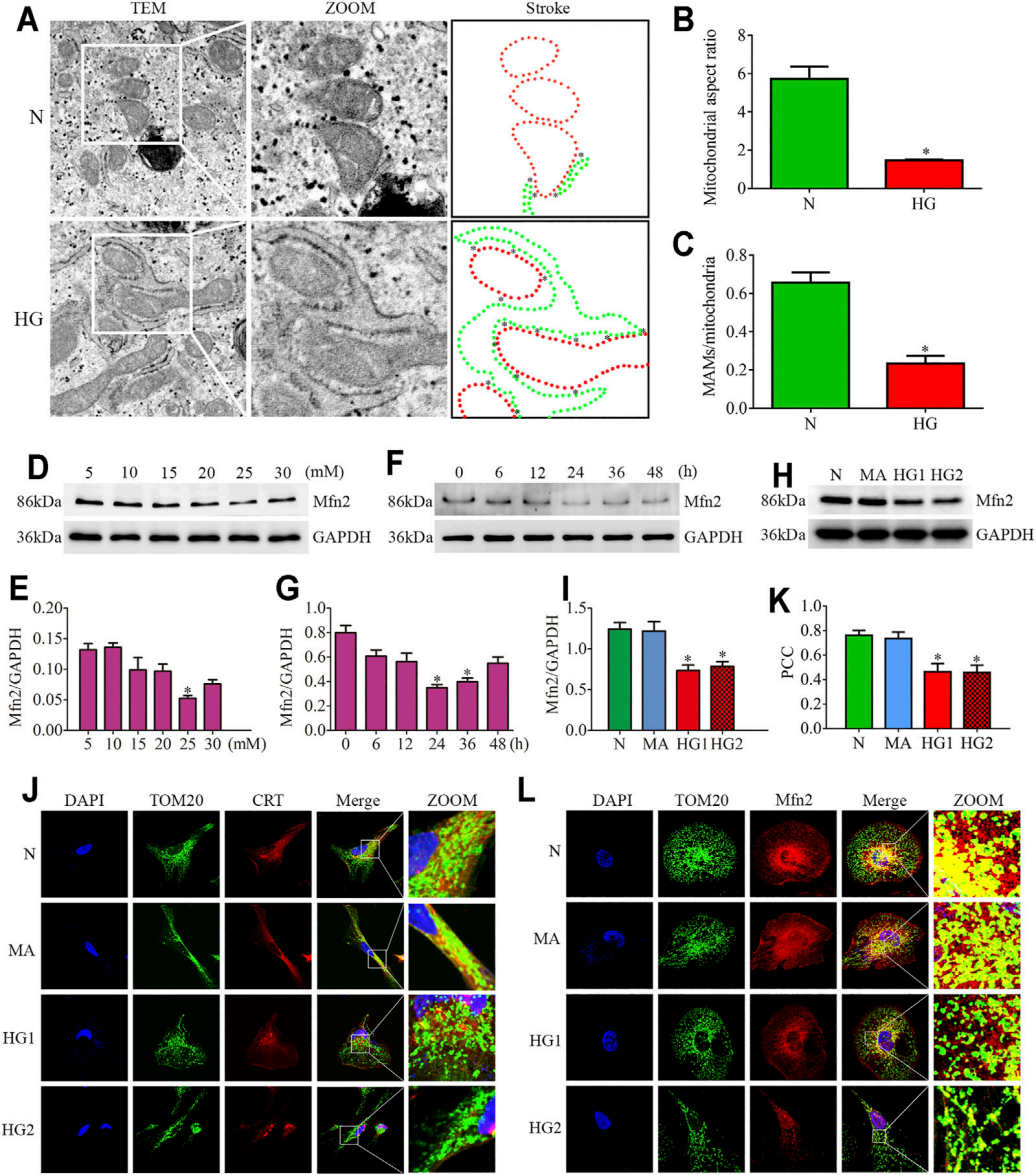
Effects of HG on MAMs and Mfn2 expression in cultured podocytes. **(A–C)** The TEM images of mitochondria, ER and MAMs in cultured podocytes and quantification of the mitochondrial aspect ratio, percentage of mitochondrial membrane in contact with ER (within a 50 nm range) to mitochondrial perimeter per group (n = 3) (original magnification, ×8,000). **(D,E)** Representative Western blots of Mfn2 expression in cultured podocytes treated with glucose concentration gradients (5, 10, 15, 20, 25, 30 mM) at 24 h and quantitation per group (n = 3). **(F,G)** Representative Western blots of Mfn2 expression in cultured podocytes treated with different time points (0, 6, 12, 24, 36, 48 h) in HG conditions (25 mM) and quantitation per group (n = 3). **(H,I)** Representative Western blots of Mfn2 expression in podocytes cultured with different medium and quantitation per group (n = 3). **(J)** Representative images of immunofluorescent staining of TOM20, CRT and DAPI in cultured podocytes per group (original magnification, ×1,000). **(K)** Quantitation of PCC of TOM20 and CRT in Figure 3J per group (n = 3). **(L)** Representative images of immunofluorescent staining of Mfn2, TOM20 and DAPI in cultured podocytes per group (original magnification, ×1,000). N = 5 mM glucose for 24h; HG = 25 mM glucose for 24h; MA = 5 mM glucose+20 mM mannitol for 24h; HG1 and HG2 = 25 mM glucose for 24 and 36h; * represent the MAMs contact points. *p < 0.05 compared with podocytes cultured in normal conditions.

The TEM photomicrographs showed increased mitochondrial fragmentation *in vivo*. Further, the loss of MAMs contact points in HPC under HG conditions was similar to the alterations in diabetic rats (Figures 3A–C). Additionally, the triple immunofluorescent staining of TOM20, CRT, and DAPI showed reduced ER-mitochondrial interactions in HG-treated HPC (Figures 3J,K).

Reduced expression of Mfn2 was also observed in HG-treated HPC. As shown in (Figures 3D–G), glucose concentration gradients (5, 10, 15, 20, 25, 30 mM) at 24 h and different time points (0, 6, 12, 24, 36, 48 h) in HG conditions (25 mM) were set to evaluate the Mfn2 expression by Western blots. The results suggested that Mfn2 expression was downregulated both in concentration-dependent and time-dependent manners under HG conditions *in vitro*. In addition, mannitol did not influence Mfn2 expression in HPC compared with normal conditions (Figures 3H,I). Furthermore, HG-treated HPC showed a dramatic decrease of Mfn2 fluorescence in the TOM20-marked mitochondria *via* immunofluorescent assays (Figure 3L). Therefore, these results suggested that HG alters the expression of Mfn2, thus, resulting in morphological and functional alterations of mitochondria and MAMs reduction in podocytes.

### Activation of PERK Pathway in Glomeruli From Diabetic Rats and HG-Treated Cultured Podocytes

Based on the previous observations linking ER stress to glomeruli damage and podocyte injury (Cybulsky, 2010), we examined the hyperglycemia or HG-induced ER stress in glomeruli from STZ-induced diabetic rats and cultured podocytes. It has been reported that the occurrence of ER stress first activates the UPR pathway, which is then marked by activation of the three sensors of UPR (IRE1, PERK, and ATF6) (Cybulsky, 2017).

**FIGURE 4:**
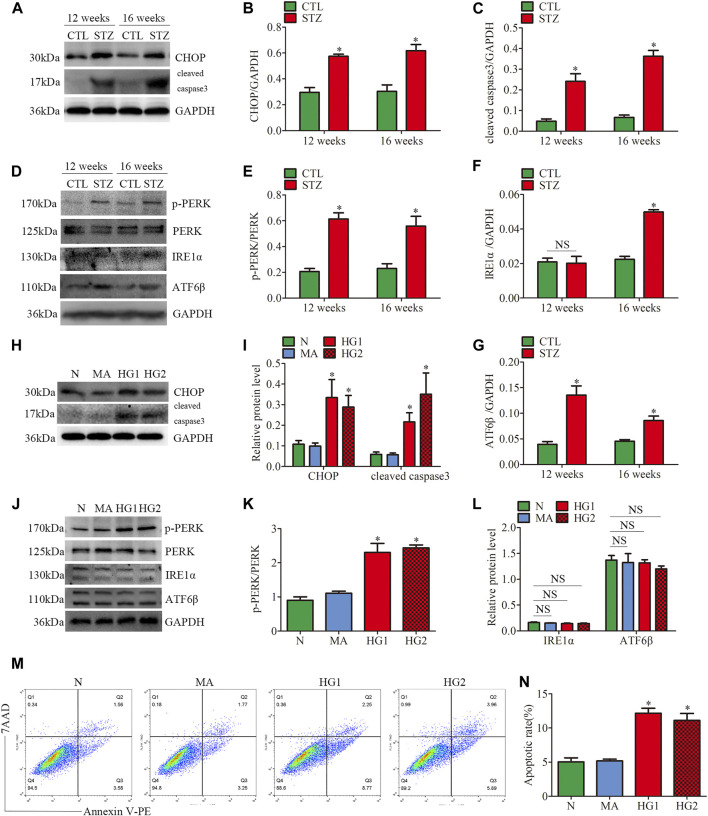
ER stress-related protein expression and apoptosis in glomeruli from diabetic rats and HG-treated cultured podocytes. **(A–G)** Representative Western blots of glomerular CHOP, cleaved caspase3, p-PERK, PERK, IRE1α, ATF6β expression and quantification per group (n = 3). *p < 0.05 relative to control. **(H–L)** Representative Western blots of CHOP, cleaved caspase3, p-PERK, PERK, IRE1α, ATF6β expression in podocytes cultured with different medium and quantitation per group (n = 3). *p < 0.05 compared with podocytes cultured in normal conditions. **(M,N)** Flow cytometry analysis of apoptosis in cultured podocytes and quantitation per group (n = 3). *p < 0.05 compared with podocytes cultured in normal conditions. CTL, control; STZ, streptozotocin; N = 5 mM glucose for 24 h; MA = 5 mM glucose+20 mM mannitol for 24 h; HG1 and HG2 = 25 mM glucose for 24 and 36 h; NS, not significant.

Firstly, the protein expression of CHOP and cleaved caspase3 was detected. CHOP is the marker of ER stress-induced apoptosis. Up-regulation of CHOP enhances apoptosis by inducting the pro-apoptotic protein Bax and suppressing the synthesis of anti-apoptotic protein Bcl2 in persistent ER stress (Hata et al., 2015). As shown in (Figures 4A–C), STZ-induced hyperglycemia markedly increased the apoptosis level of glomeruli, which was consistent with our previous observation (Fan et al., 2019; Lin et al., 2020). Decreased Bcl2 expression and increased Bax expression were also observed in glomeruli from diabetic rats (Supplementary Figures 3A–C). Subsequently, we observed the protein levels of three UPR sensors (p-PERK, IRE1α, ATF6β) in glomeruli from STZ-treated rats. Compared with the controls, the diabetic rats had significantly increased expression of p-PERK, IRE1α and ATF6β (Figures 4D–G). These findings suggested that the three UPR signaling pathways were activated in glomeruli from STZ-treated rats. In addition, increased podocyte apoptosis in diabetic rats was observed by the triple immunofluorescent staining of TUNEL, Synaptopodin, and DAPI (Supplementary Figure 3G).

Consistent with the results of the *in vivo* experiments, the HG-treated HPC showed increased levels of apoptosis-related proteins CHOP and cleaved caspase3 (Figures 4H,I). HG treatment also suppressed Bcl2 expression and induced Bax expression *in vitro* (Supplementary Figures 3D–F). Different from the results of the *in vivo* experiments, the increased p-PERK/PERK protein ratio was detected in HG-treated HPC, while no significant changes in the expression of IRE1α and ATF6β (Figures 4J–L). Thus, the results indicated that only the PERK pathway was activated in HG-treated podocytes. Moreover, flow cytometry examined cell apoptosis and showed that HG significantly provokes podocyte apoptosis (Figures 4M, N). These findings demonstrated that ER stress was associated with the progression of glomerular damage and podocyte injury in DKD, and activation of the PERK pathway may be the key factor of podocyte apoptosis in DKD.

### Effects of HG on the Mitochondrial Dysfunction and Mfn2-PERK Interaction in Glomeruli From Diabetic Rats and HG-Treated Cultured Podocytes

**FIGURE 5:**
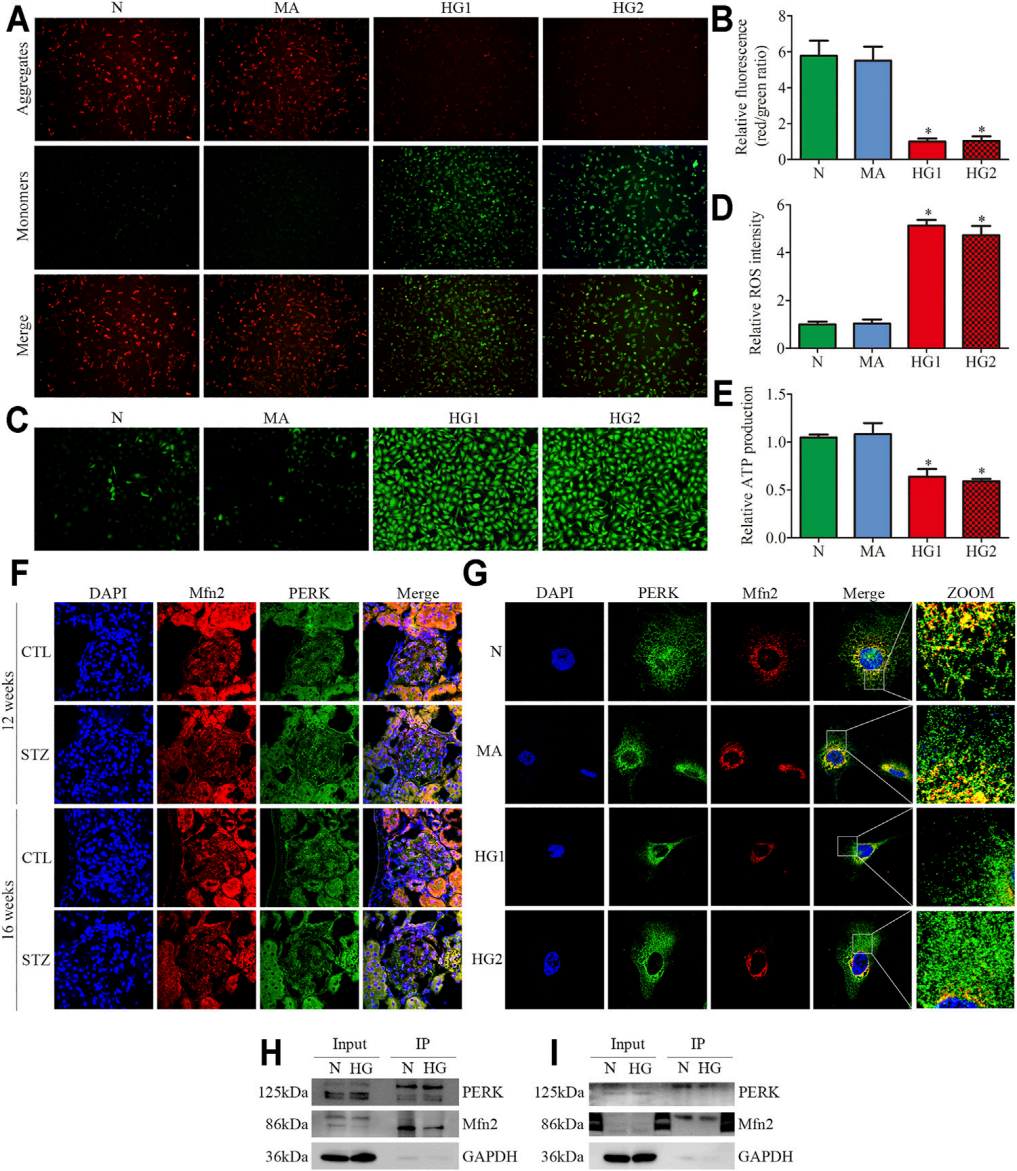
Mitochondrial functions and Mfn2-PERK interaction in glomeruli from diabetic rats and HG-treated cultured podocytes. **(A,B)** Representative images of MMP in cultured podocytes by JC-1 staining and quantification per group (n = 3) (original magnification, ×100). **(C,D)** Representative images of DCFH-DA fluorescence (green) in cultured podocytes to assess ROS production and quantification per group (n = 3) (original magnification, ×100). **(E)** ATP production was assessed and quantification per group (n = 3). **(F,G)** Representative images of immunofluorescent staining of Mfn2, PERK and DAPI in glomeruli and cultured podocytes per group (original magnification,×600, ×1,000). **(H,I)** Representative coimmunoprecipitation results of the interaction between Mfn2 and PERK per group (H or I) Podocyte lysates were subjected to immunoprecipitation with PERK or Mfn2 antibody then the immunoprecipitates were blotted with Mfn2 or PERK antibody. CTL, control; DKD, diabetic kidney disease; N = 5 mM glucose for 24 h; MA = 5 mM glucose+20 mM mannitol for 24 h; HG1 and HG2 = 25 mM glucose for 24 and 36 h; HG (Co-IP) = 25 mM glucose for 24 h; *p < 0.05 compared with podocytes cultured in normal conditions.

JC-1 staining, DCFH-DA fluorescent probe, and ATP assay were conducted to further explore the HG-induced alterations in mitochondrial functions of podocytes. The mitochondrial membrane potential (MMP) is one of the indicators of mitochondrial functions. JC-1 staining revealed that HG exposure markedly decreased MMP (Figures 5A,B). DCFH-DA fluorescent probe was used to detect cellular oxidative stress, as shown in (Figures 5C,D), intracellular ROS generation was markedly increased in the HG-treated HPC. Furthermore, the ATP production decreased in HPC under HG conditions (Figure 5E). These results suggested that HG stimulation negatively affects mitochondrial functions.

Recent studies have revealed that PERK is located at the same membranes (MAMs) as Mfn2 (Verfaillie et al., 2012; Lebeau et al., 2018). Under basal conditions, Mfn2-PERK interaction keeps PERK in an inactive form, which is essential for maintaining ER homeostasis. The triple immunofluorescent staining (Mfn2, PERK, and DAPI) exhibited reduced colocalization of Mfn2 and PERK in glomeruli from diabetic rats and HG-treated HPC (Figures 5F,G). The co-immunoprecipitation assay revealed that Mfn2 physically interacts with PERK in HPC, which was in line with previous studies (Munoz et al., 2013). Additionally, HG treatment induced a dramatic reduction in Mfn2-PERK interaction (Figures 5H,I). These results indicated that HG activated the PERK pathway by downregulating Mfn2 expression; thus, reducing Mfn2-PERK interaction. Therefore, we speculated that the downregulation of Mfn2 expression might result in Mfn2-PERK interaction dissociating and PERK pathway activating in HG-treated podocytes. Subsequently, leading to MAMs reduction and mitochondrial dysfunction.

### Inhibition of PERK Phosphorylation Attenuated HG-Induced Mitochondrial Dysfunction and MAMs Reduction

**FIGURE 6:**
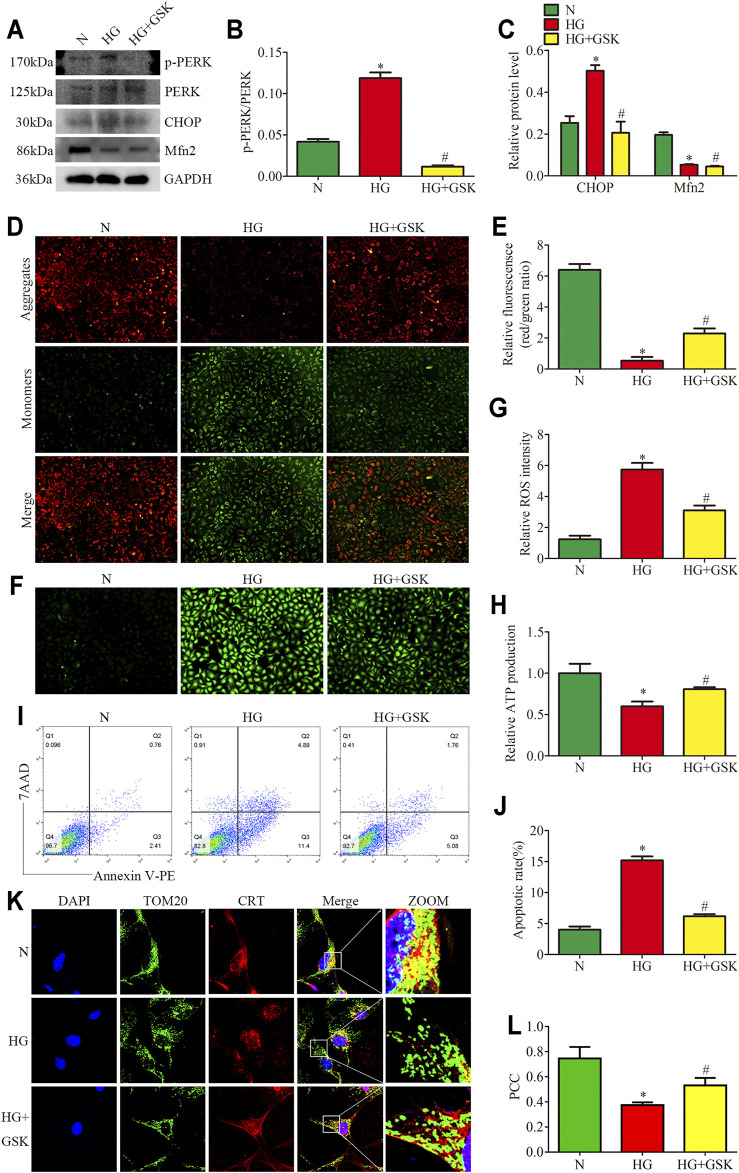
Effects of PERK inhibition on HG-induced mitochondrial dysfunction and MAMs reduction. **(A–C)** Representative Western blots of p-PERK, PERK, CHOP, Mfn2 expression and quantitation per group (n = 3). **(D,E)** Representative images of MMP in cultured podocytes by JC-1 staining and quantification per group (n = 3) (original magnification, ×100). **(F,G)** Representative images of DCFH-DA fluorescence (green) in cultured podocytes to assess ROS production and quantification per group (n = 3) (original magnification, ×100). **(H)** ATP production was assessed and quantification per group (n = 3). **(I,J)** Flow cytometry analysis of apoptosis in cultured podocytes and quantitation per group (n = 3). **(K)** Representative images of immunofluorescent staining of TOM20, CRT and DAPI in cultured podocytes per group (original magnification, ×1,000) **(L)** Quantitation of PCC of TOM20 and CRT in Figure 6K per group (n = 3). N = 5 mM glucose for 24 h; HG = 25 mM glucose for 24 h; HG + GSK = 25 mM glucose and 20 μM GSK2656157 for 24 h; NS, not significant; *p < 0.05 compared with podocytes cultured in normal conditions; #*p* < 0.05 compared with podocytes treated with HG.

To assess the regulatory effects of the PERK pathway on mitochondrial functions and MAMs *in vitro*, the HPC were treated with PERK inhibitor (GSK2656157; 20μM, 24 h) in the presence of HG (25 mM, 24 h). We found that the treatment with GSK blocked PERK phosphorylation and downregulated the CHOP expression in HPC under HG conditions, but exhibited no effects on Mfn2 protein level (Figures 6A–C). GSK treatment also reversed HG-induced Bcl2 and Bax expression *in vitro* (Supplementary Figures 4A–C). Additionally, as shown in (Figures 6K, 6L), increased co-localization of TOM20 and CRT after GSK treatment was detected under HG conditions. Moreover, GSK attenuated the HG-induced mitochondrial dysfunction and apoptosis in HPC by restoring the MMP, reducing ROS generation, and up-regulating the ATP levels (Figures 6D–J). These results indicated that Mfn2 may lay upstream of PERK to regulate MAMs and mitochondrial functions.

### Mfn2 Regulated PERK Pathway Related-Mitochondrial Dysfunction, MAMs Reduction, and Apoptosis Under HG Conditions

**FIGURE 7:**
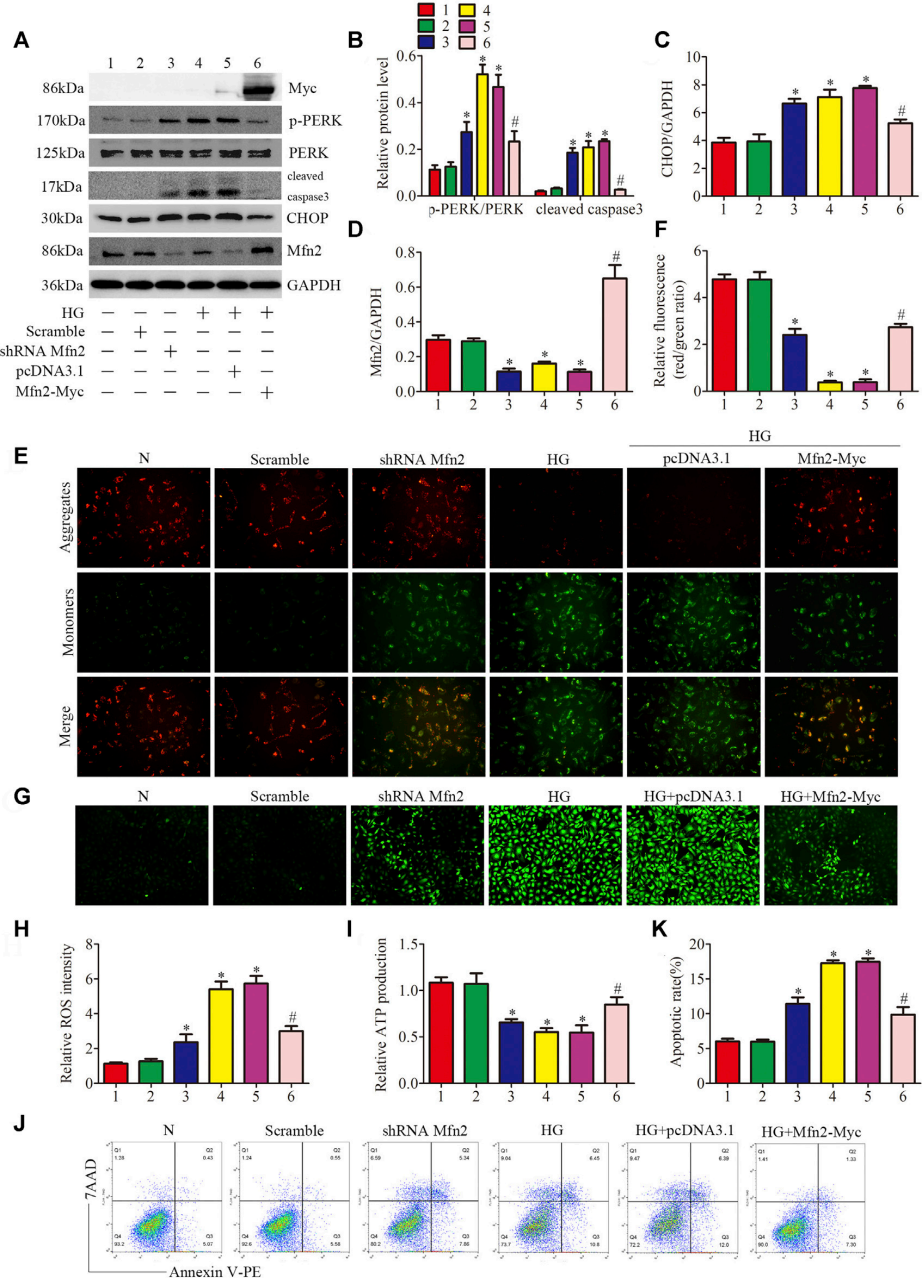
Role of Mfn2 in PERK signaling pathway related-mitochondrial dysfunction, MAMs reduction and apoptosis. **(A–D)** Representative Western blots of Myc-tag, p-PERK, PERK, cleaved caspase3, CHOP, Mfn2 expression and quantitation per group (n = 3). **(E, F)** Representative images of MMP in cultured podocytes by JC-1 staining and quantification per group (n = 3) (original magnification, ×200). **(G,H)** Representative images of DCFH-DA fluorescence (green) in cultured podocytes to assess ROS production and quantification per group (n = 3) (original magnification, ×100). **(I)** ATP production was assessed and quantification per group (n = 3). **(J, K)** Flow cytometry analysis of apoptosis in cultured podocytes and quantitation per group (n = 3). 1, 2, 3, 4, 5, 6 represent N, Scramble, shRNA Mfn2, HG, HG + pcDNA3.1, HG + Mfn2-Myc, respectively; N = 5 mM glucose for 24 h; HG = 25 mM glucose for 24 h; Scramble = shRNA-scrambles; shRNA = short hairpin RNA; *p < 0.05 compared with podocytes cultured in normal conditions; #*p* < 0.05 compared with podocytes treated with HG.

Based on the above data, we identified Mfn2-PERK interaction as a potential site influenced by HG, and Mfn2 may regulate mitochondrial functions and MAMs through the PERK pathway. To further examine the underlying mechanisms, Mfn2-Myc plasmid or shRNA Mfn2 plasmid were transfected into the HPC to overexpress or silence Mfn2. The transfection of Mfn2-Myc plasmid reduced the phosphorylation of PERK and the expression of apoptosis-related proteins (cleaved caspase3 and CHOP), reversed the downregulation of Mfn2 in HG-treated HPC. Conversely, shRNA Mfn2 plasmid transfection increased the phosphorylation of PERK along with the upregulated expression of cleaved caspase3 and CHOP under normal conditions (Figures 7A–D). Consistent with previous speculations, Mfn2 overexpression restored Mfn2-PERK interaction, while Mfn2 silencing reduced the interaction under normal conditions (Supplementary Figure 5C).

The function of podocytes is largely based on maintaining the normal actin-based foot processes structure (He et al., 2013). As shown in (Supplementary Figure 5D), exposure to HG or Mfn2 silencing induced rearrangement of the actin cytoskeleton in HPC, which was prevented by the Mfn2 overexpression. Furthermore, overexpression of Mfn2 attenuated HG-induced mitochondrial dysfunction and apoptosis compared with the HG group. In contrast, Mfn2 silencing triggered mitochondrial dysfunction and apoptosis under normal conditions (Figures 7E–K). Moreover, the immunofluorescence staining showed that the HG + Mfn2-Myc group restored TOM20-CRT interaction while the shRNA Mfn2 group decreased the interaction (Supplementary Figures 5A,B). Taken together, these results demonstrated that Mfn2 plays a protective and anti-apoptotic role in protecting mitochondria and MAMs via suppressing the activation of the PERK pathway.

## Discussion

Podocyte injury and loss are considered to be the key events in the progression of DKD. ER stress and mitochondrial dysfunction play vital roles in podocyte injury and apoptosis in the diabetic kidney. However, the molecular mechanisms have not been defined completely. Thus, finding the key molecule involved in ER homeostasis and mitochondrial regulation in podocytes will be a promising therapeutic target for DKD.

**FIGURE 8:**
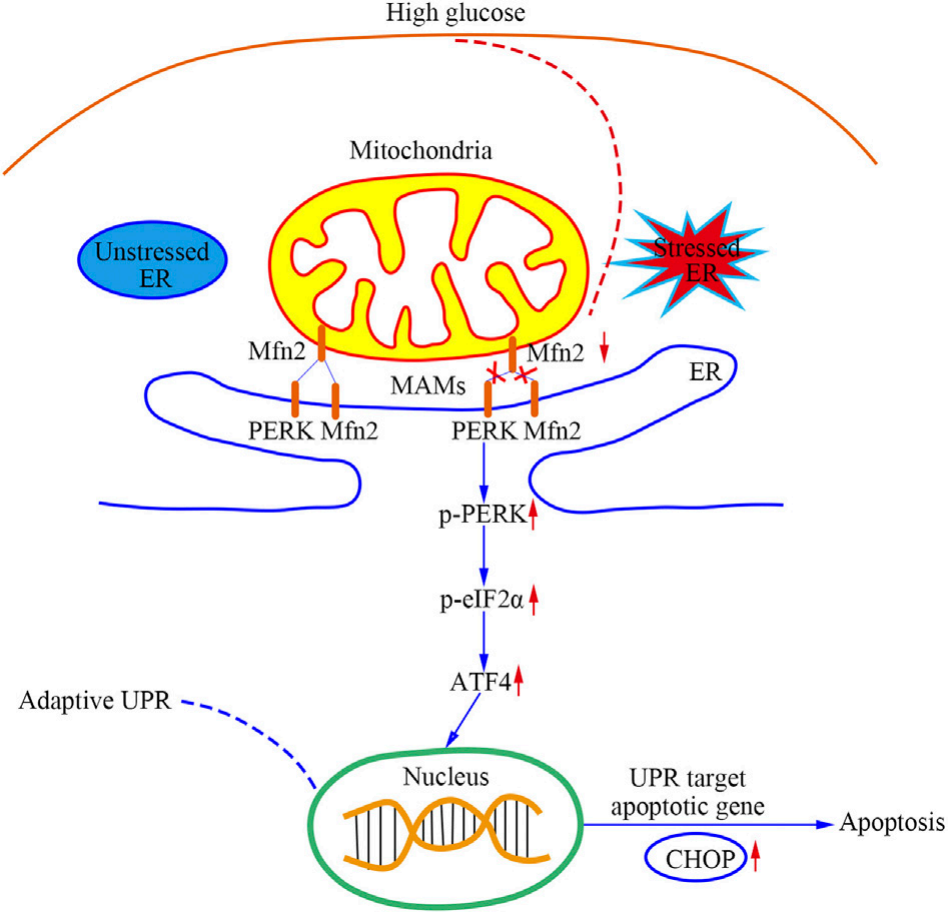
A schematic summary of the effects of Mfn2-PERK signaling pathway on podocyte injury and apoptosis caused by HG-induced MAMs and mitochondrial dysfunction.

As shown in Figure 8, this study provided evidence that a PERK pathway-related mechanism is responsible for developing mitochondrial dysfunction, MAMs reduction, and apoptosis in Mfn2-downregulated podocytes under HG conditions. Our findings are described as follows: 1) Podocytes under HG conditions exhibited decreased Mfn2 expression accompanied by mitochondrial morphological alterations and dysfunction, reduced MAMs, and increased apoptosis both *in vivo* and *in vitro*; 2) Podocytes under HG conditions showed proapoptotic protein upregulation and PERK pathway activation both *in vivo* and *in vitro*; 3) Mfn2 interacted PERK physically in podocytes; 4) Inhibition of PERK phosphorylation ameliorated mitochondrial dysfunction, MAMs reduction and apoptosis, but it did not affect Mfn2 expression; 5) Mfn2 silencing activated the PERK pathway, reduced MAMs, provoked mitochondrial dysfunction and apoptosis under normal conditions; 6) Overexpression of Mfn2 suppressed PERK pathway activation, attenuated the MAMs reduction, mitochondrial dysfunction, and apoptosis under HG conditions.

Mitochondria is a highly dynamic tubular organelle undergoing continuous remodeling. Increasing evidence suggested that mitochondrial morphology is closely linked to functions (Giacomello et al., 2020; Rovira-Llopis et al., 2017). MAMs is a dynamic platform consisting of multiple proteins that maintain stable spacing between ER membrane and OMM without fusion between two membranes. Mfn2 has been widely acknowledged as the vital OMM and ER-mitochondrial tethering component in different cell types (Gbel et al., 2020; Li et al., 2015). Han et al. reported that Mfn2 ablation reduced the MAMs, whereas Mfn2 overexpression increased close contacts between ER and mitochondria *in vivo* (Han et al., 2021). This study used different samples and methods to explore the role of Mfn2 in mitochondria and MAMs of podocytes. In par with previous reports, we observed that HG-induced Mfn2 downregulation accompanied by increased small spherical mitochondria and decreased MAMs contact points. These data supported that Mfn2 plays an essential role in maintaining mitochondrial morphology and ER-mitochondria tethering.

However, more recent studies challenged the tethering effect of Mfn2 on the ER-mitochondrial contact sites since the mutation or deletion of Mfn2 increased the coupling between the ER and mitochondria (Filadi et al., 2015; Leal et al., 2016). The difference might be dependent on the cell type and the observation time after Mfn2 KO. Thus, further investigations are necessary to evaluate the detailed impact of Mfn2 in MAMs.

Vance et al. first separated and characterized the MAMs fraction (Vance, 1990). The composition of MAMs determines that it's essential role in mitochondrial and ER functions, including ER stress. This study observed that ER stress was activated after the HG-induced Mfn2 downregulation *in vivo* and *in vitro*. Furthermore, Mfn2-downregulated podocytes showed an exaggerated activation of the PERK pathway. This study also verified that the Mfn2-PERK interaction reduction and apoptosis accompanied Mfn2 downregulation in line with previous studies, which reconfirmed that Mfn2 also interact with PERK and keep it inactive in the podocytes under basal conditions. The up-regulation of UPR-related apoptotic proteins could explain HG and Mfn2 silencing-induced podocyte apoptosis. In ER stress, the imbalance of Ca^2+^ homeostasis is an essential pathway of MAMs-related apoptosis. Ca^2+^ in the ER flows into the mitochondria through Ca^2+^ channels of MAMs, which further disturbs energy metabolism and the regular activity of cells consequently (Li et al., 2021). Impaired Ca^2+^ flux between ER and mitochondria also induces activation of the NLRP3 inflammasome (Missiroli et al., 2018). The NLRP3 inflammasome has been proved that recruited and activated upon the MAMs (Zhang et al., 2017). Moreover, Mfn2-UPR interaction plays an important regulatory role in inflammation response. For instance, overexpression of Mfn2 alleviated TNFα-mediated ER stress in N2a cells by suppressing the expression of CHOP, caspase3, and caspase12 (Hou et al., 2019).

The UPR maintains regular activity and survival of cells at first are called the adaptive UPR. By contrast, prolonged UPR may be cytotoxic when ER homeostasis cannot reestablish. This study found that the PERK/eIF2α/ATF4 pathway is closely related to HG-induced downregulation of Mfn2. The PERK/eIF2α/ATF4 pathway activation inhibits global protein synthesis and drives the pro-apoptosis (Athanasiou et al., 2017). CHOP is the UPR-induced apoptosis marker and is activated by the PERK pathway mainly. Up-regulation of CHOP enhances apoptosis by inducting the proapoptotic protein such as Bax, Bak and suppressing the synthesis of anti-apoptotic protein such as Bcl2 and Bcl-xl in persistent UPR (Hata et al., 2015). UPR in DKD has been well-documented. Earlier studies found that the STZ-induced DM rats showed increased levels of GRP78, p-PERK, ATF4 and CHOP in kidneys, as well as enhanced apoptosis (Yuan et al., 2018). In addition to the PERK pathway, connections between Mfn2 and the other two UPR pathways also existed in this study. Recent studies also reported that podocyte-specific IRE1α or X box-binding protein1 (XBP1)-KO mice exhibited podocyte injury and affected UPR (Hassan et al., 2016; Kaufman et al., 2017). The DKD aggravated in transgenic mice overexpressing ATF6 in podocytes (Madhusudhan et al., 2015). In our study, PERK phosphorylation inhibitor ameliorated HG-induced mitochondrial morphological alterations and dysfunction, reduced MAMs and increased apoptosis, but did not influence Mfn2 expression. The results provided a basis for verification that Mfn2 is the upstream regulator of PERK.

There are also some limitations to our results and list as follows: 1) Renal biopsies from patients with DKD according to DKD staging could further analyze the role of Mfn2 in the development of DKD; 2) Lack of a podocyte-specific Mfn2 knockout animal model to directly observe the effect of Mfn2 deletion on PERK pathway-related alterations in podocytes; 3) Lack of functional testing of MAMs such as Ca^2+^ signal transmission to provide a more complete theoretical basis for our conclusion.

In conclusion, this study validate that Mfn2 mediates mitochondrial dysfunction, MAMs reduction, and podocytes apoptosis through activating the PERK pathway. It also provides important evidence that targeting the Mfn2-PERK signaling pathway may serve as a therapeutic strategy for HG-induced podocyte injury and apoptosis in DKD.

## Data Availability

The original contributions presented in the study are included in the article/Supplementary Material, further inquiries can be directed to the corresponding author.
