# Supplementary figures and images for: Mfn2 Regulates High Glucose-Induced MAMs Dysfunction and Apoptosis in Podocytes via PERK Pathway
Source: Front Cell Dev Biol. 2021 Dec 20;9:769213. doi: 10.3389/fcell.2021.769213 (PMC8721005; doi:10.3389/fcell.2021.769213)

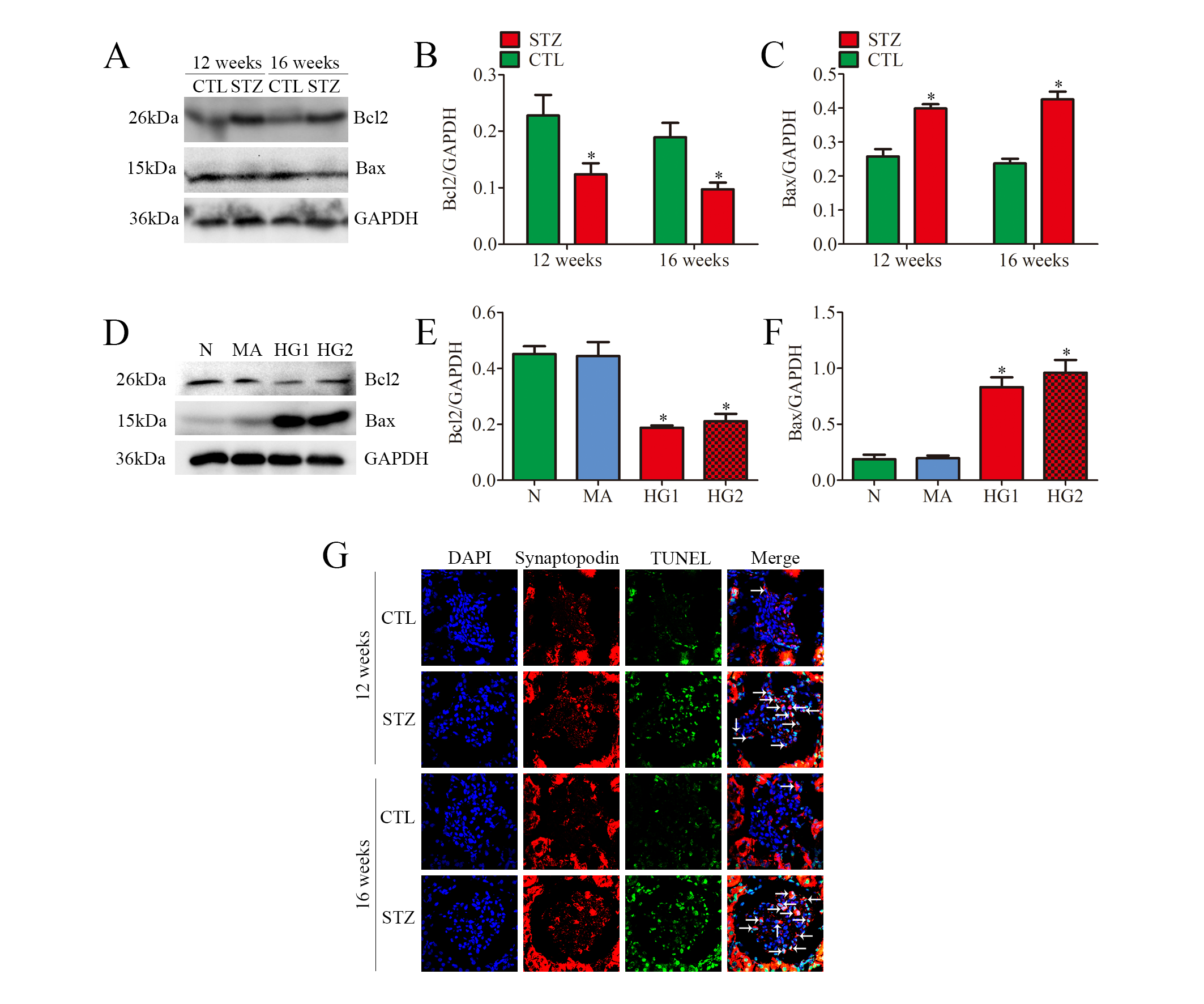

Supplement: Supplementary file 1 [file Image3.TIF]

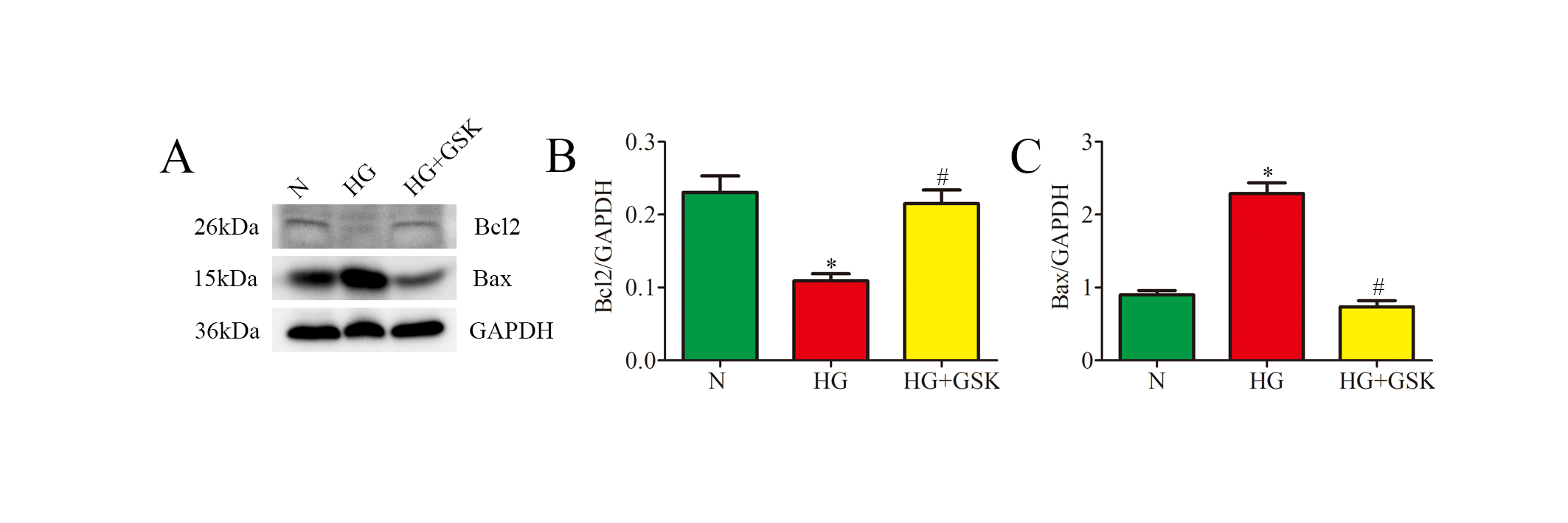

Supplement: Supplementary file 2 [file Image4.TIF]

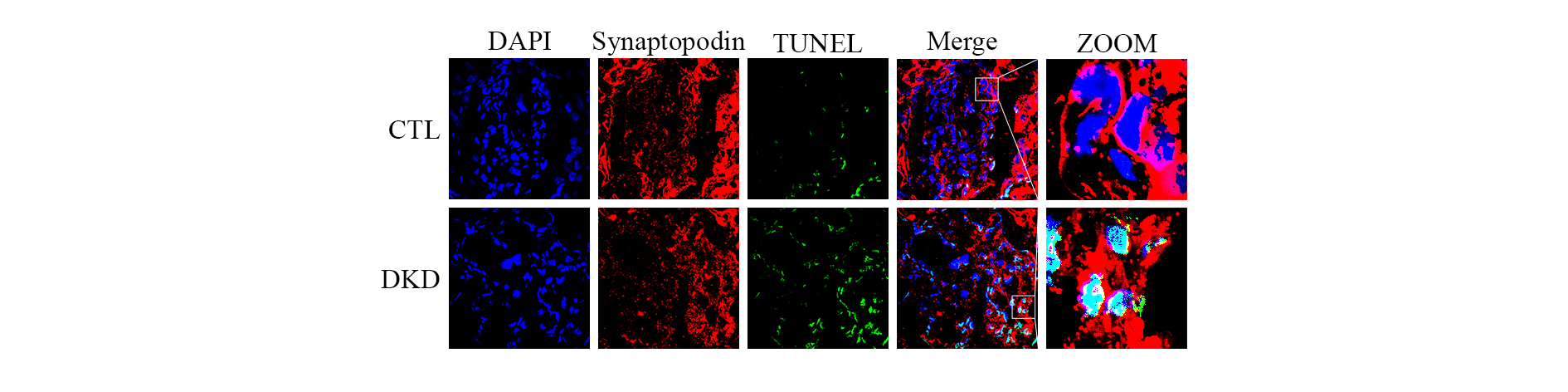

Supplement: Supplementary file 3 [file Image2.TIF]

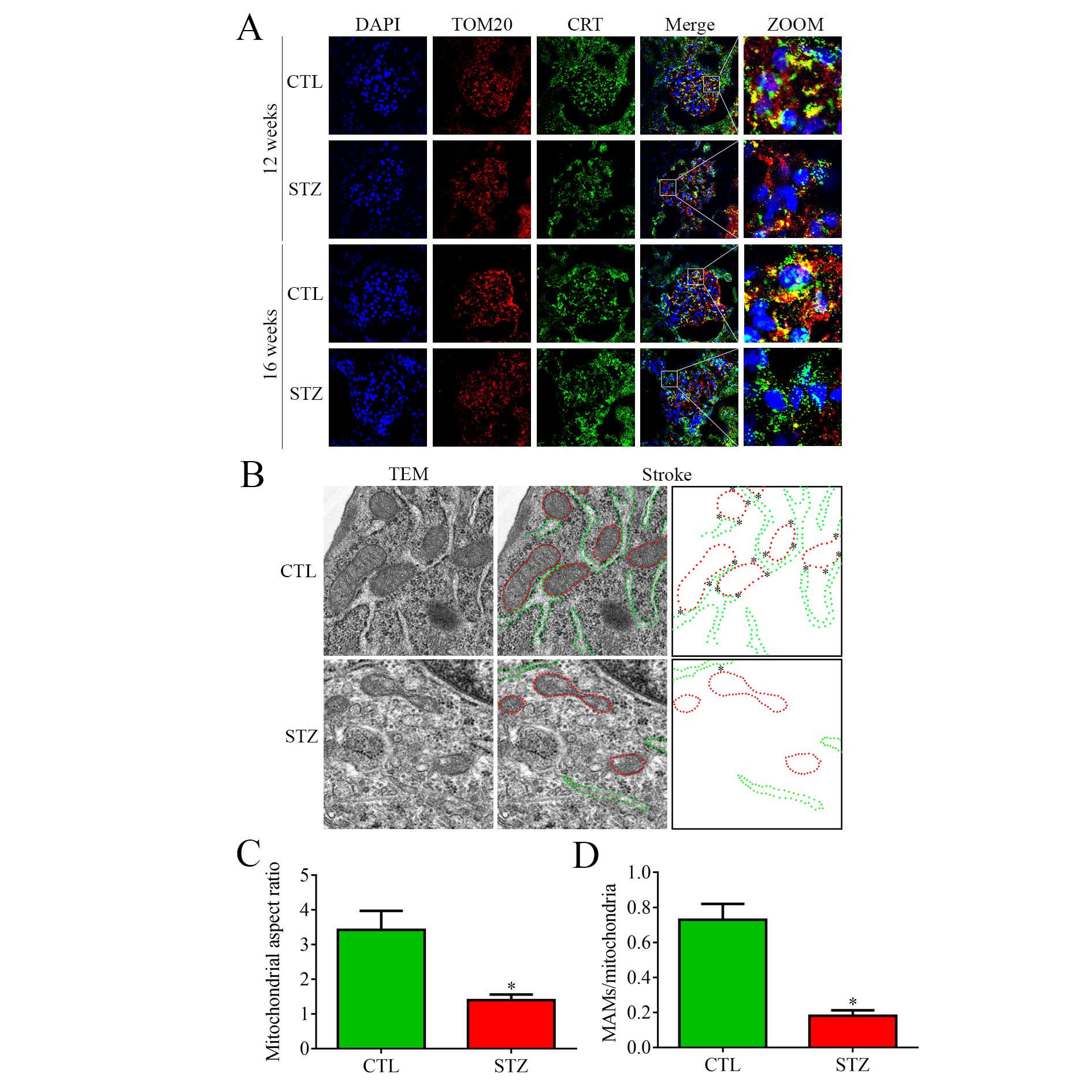

Supplement: Supplementary file 4 [file Image1.TIF]

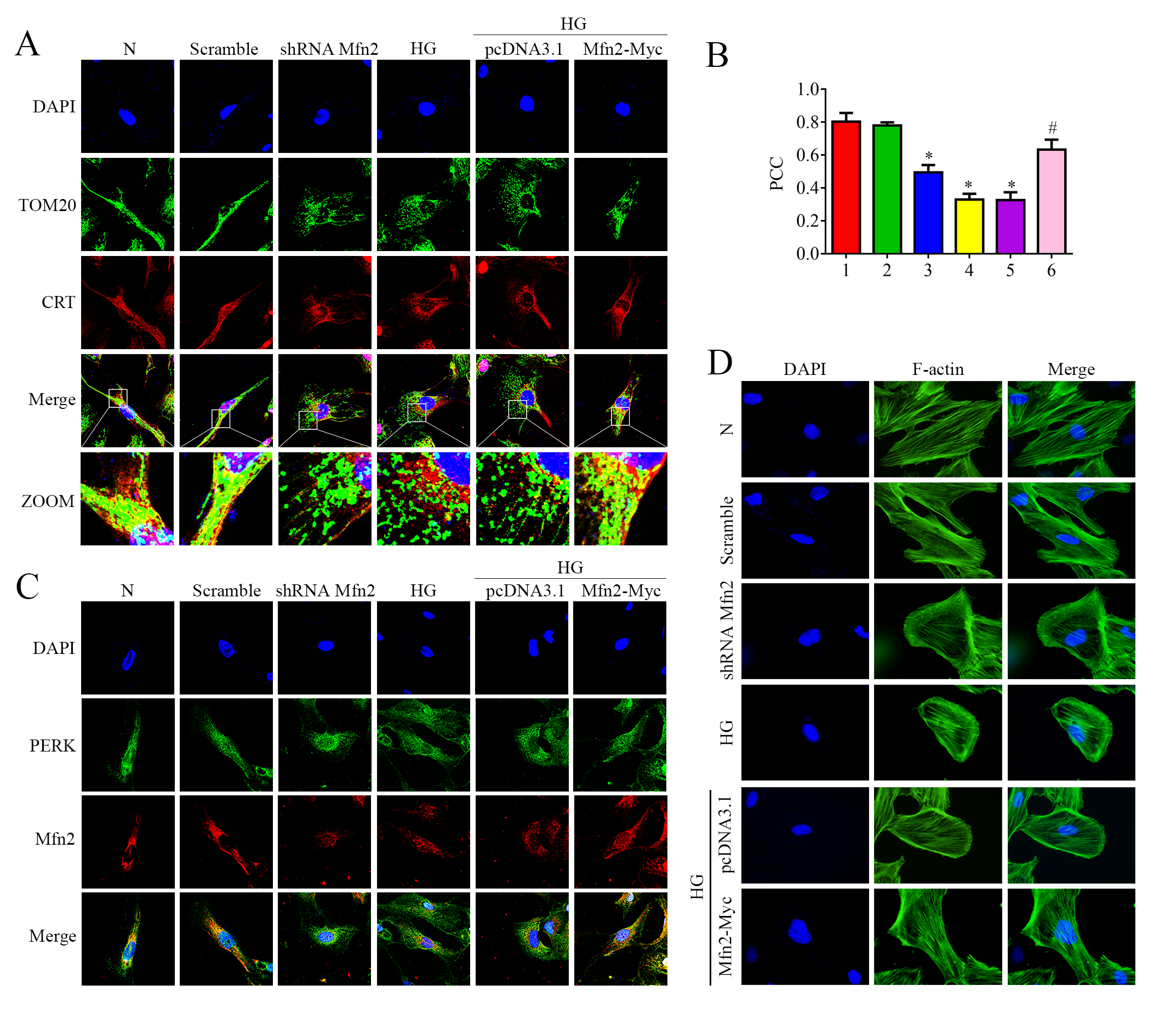

Supplement: Supplementary file 5 [file Image5.TIF]
