# Supplementary material for: Mfn2 Regulates High Glucose-Induced MAMs Dysfunction and Apoptosis in Podocytes via PERK Pathway
Source: Front Cell Dev Biol. 2021 Dec 20;9:769213. doi: 10.3389/fcell.2021.769213 (PMC8721005; doi:10.3389/fcell.2021.769213)
Supplement: Supplementary file 6 [file DataSheet1.docx]

**Supplement**

**Materials and methods**

**Phalloidin staining**

Phalloidin is an F-actin marker. The phalloidin staining using the cell climbing films were performed. After washing, the samples were fixed with 4% paraformaldehyde for 15 min, and then were incubated with 100μL phalloidin (1:1000; Sigma‐Aldrich, USA) working fluid in the dark for 1h at 37°C. The nuclei were stained with an anti-fluorescence quencher containing DAPI (Antgene, Wuhan, China). An upright microscope (Olympus, Japan) was used to observe all of the microscopic images.

**Supplementary Figure 1. MAMs alterations of glomeruli and podocytes in diabetic rats**


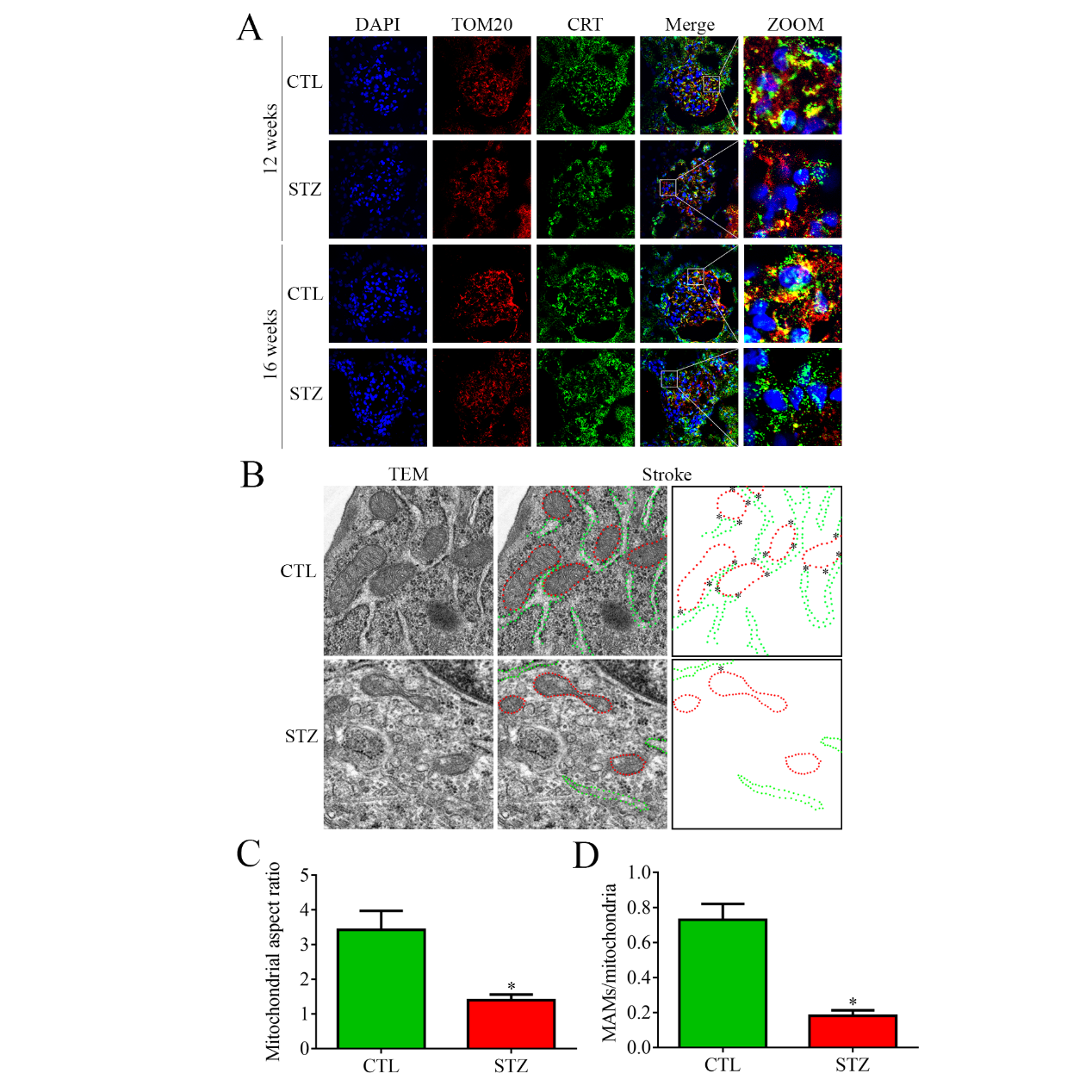
(A) Representative images of immunofluorescent staining of TOM20, CRT and DAPI in glomeruli per group (original magnification, ×600). (B-D) The TEM images of mitochondria, ER and MAMs in glomerular podocytes, and then quantification of the mitochondrial aspect ratio, percentage of mitochondrial membrane in contact with ER (within a 50nm range) to mitochondrial perimeter per group (n=3) (original magnification, ×8000). CTL, control; STZ, streptozotocin. * represent the MAMs contact points. *p<0.05 relative to control.

**Supplementary Figure 2. Podocyte apoptosis in patients with DKD**


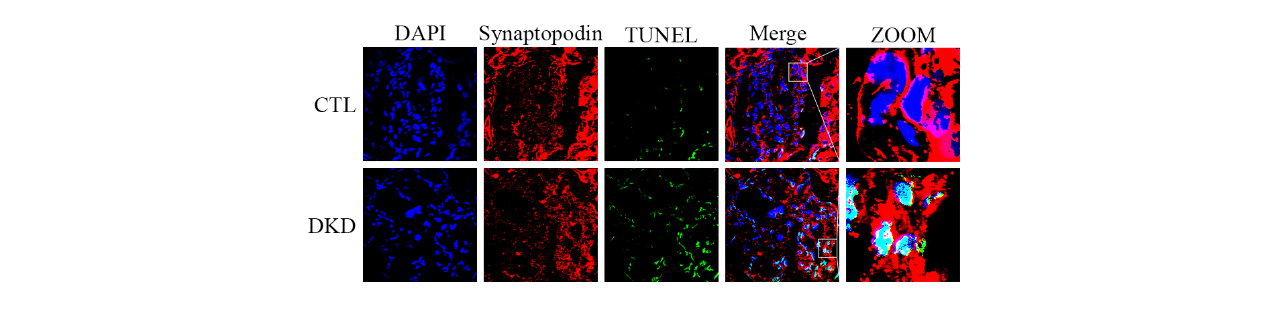


Representative images of immunofluorescent staining of TUNEL, Synaptopodin and DAPI in glomeruli per group (original magnification, ×600). CTL, control; DKD, diabetic kidney disease.

**Supplementary Figure 3. Apoptosis of podocytes in diabetic rats and HG-treated cultured podocytes**


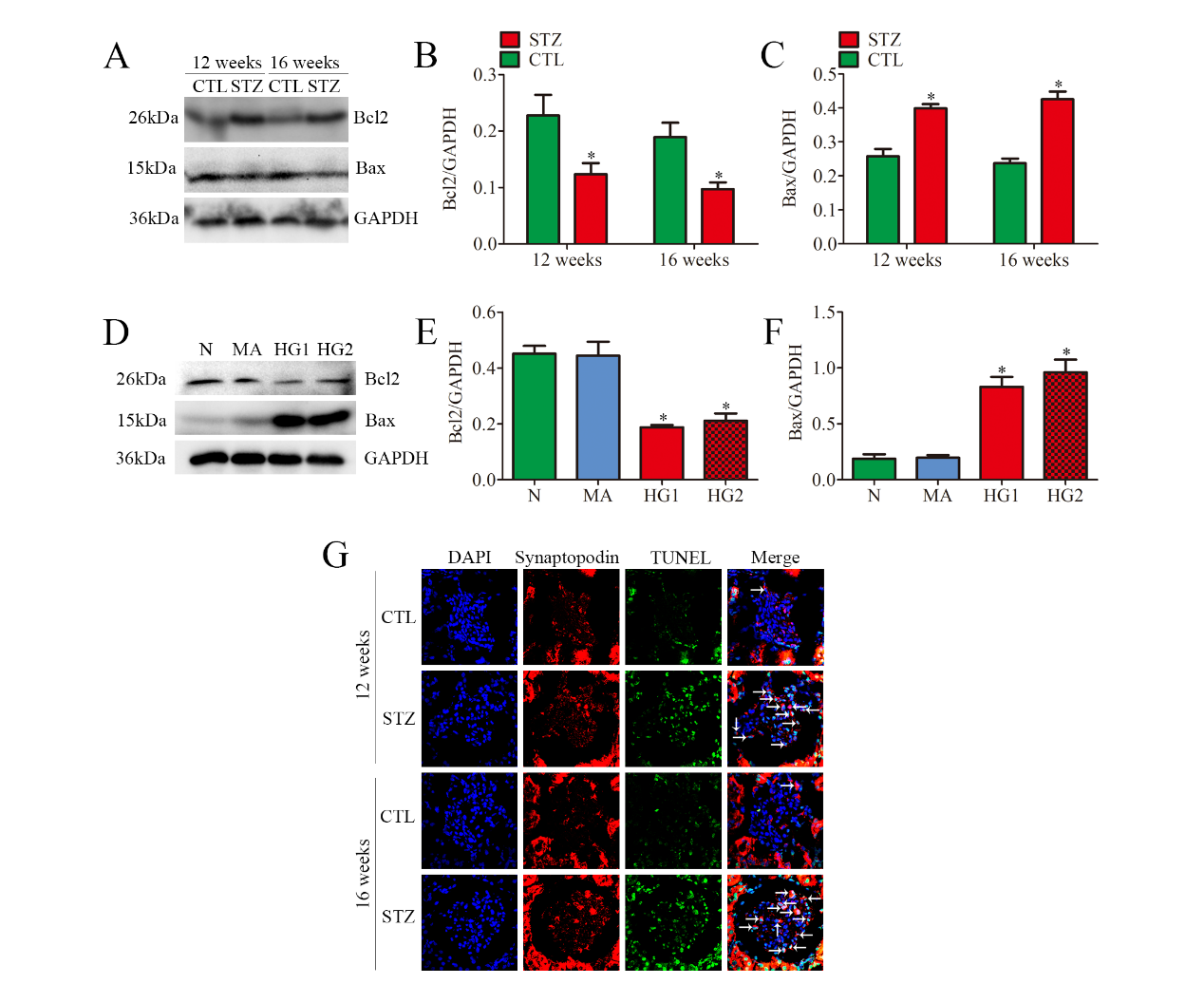


(A-C) Representative Western blots of glomerular Bcl2 and Bax expression and quantitation per group (n=3). *p<0.05 relative to control. (D-F) Representative Western blots of Bcl2 and Bax expression in podocytes cultured with different medium and quantitation per group (n=3). *p<0.05 compared with podocytes cultured in normal conditions. (G) Representative images of immunofluorescent staining of TUNEL, Synaptopodin and DAPI in glomeruli per group (original magnification, ×600), Apoptotic cells are indicated by arrows. CTL, control; STZ, streptozotocin; N=5mM glucose for 24h; MA=5mM glucose+20mM mannitol for 24h; HG1 and HG2=25mM glucose for 24h and 36h.

**Supplementary Figure 4.** **Effects of PERK inhibition on apoptosis of HG-treated cultured podocytes**


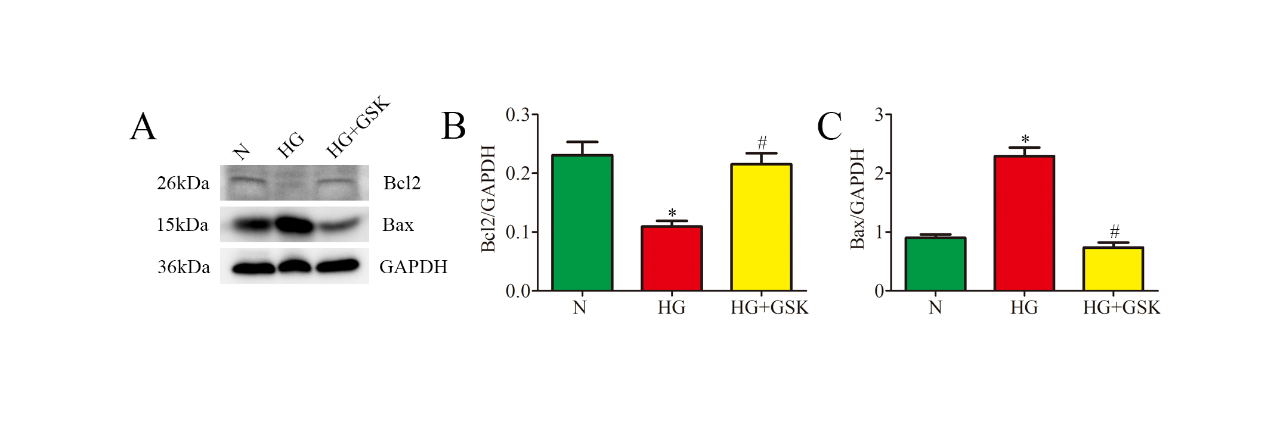


(A-C) Representative Western blots of Bcl2 and Bax expression and quantitation per group (n=3). N=5mM glucose for 24h; HG=25mM glucose for 24h; HG+GSK=25mM glucose and 20µM GSK2656157 for 24h. *p<0.05 compared with podocytes cultured in normal conditions; #p<0.05 compared with podocytes treated with HG.

**Supplementary Figure 5.** **Role of Mfn2 in MAMs and actin cytoskeleton of cultured podocytes**


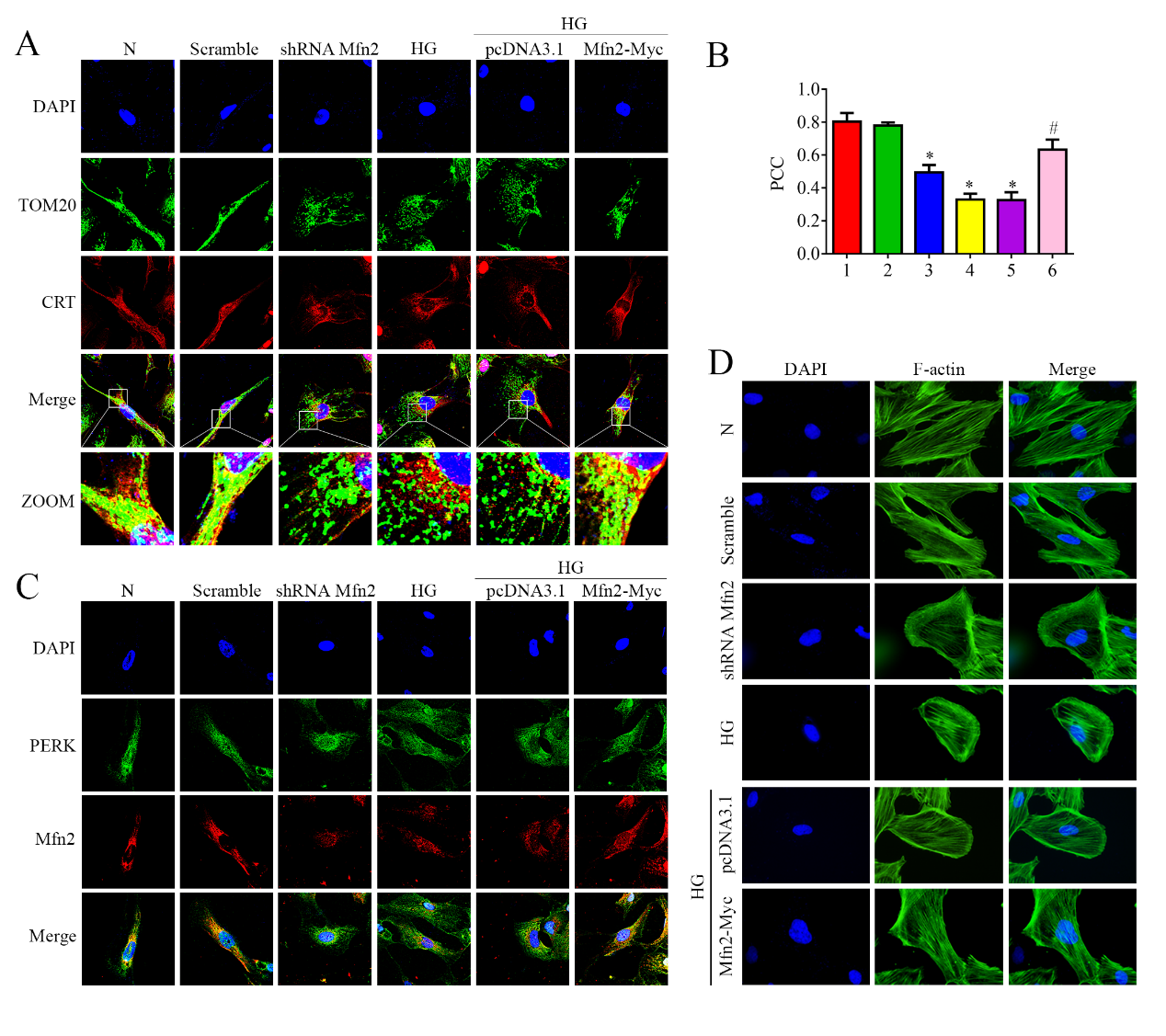


(A) Representative images of immunofluorescent staining of TOM20, CRT and DAPI in cultured podocytes per group (original magnification, ×1000); (B) Quantitation of PCC of TOM20 and CRT in Supplementary Figure 5A per group (n=3). (C) Representative images of immunofluorescent staining of Mfn2, PERK and DAPI in cultured podocytes per group (original magnification, ×1000). (D) Representative images of immunofluorescent staining of F-actin (phalloidin staining) and DAPI in cultured podocytes per group (original magnification, ×1000). 1, 2, 3, 4, 5, 6 represent N, Scramble, shRNA Mfn2, HG, HG+pcDNA3.1, HG+Mfn2-Myc, respectively; N=5mM glucose for 24h; HG=25mM glucose for 24h; Scramble=shRNA-scrambles; shRNA=short hairpin RNA. *p<0.05 compared with podocytes cultured in normal conditions; #p<0.05 compared with podocytes treated with HG.
